# Fracture Topology in Mafic Formations: Implications for Geological Carbon Storage

**DOI:** 10.1021/acs.energyfuels.5c03097

**Published:** 2025-09-09

**Authors:** Andrea Muñoz-Ibáñez, J. Carlos Santamarina

**Affiliations:** † 1372 Georgia Institute of Technology, Atlanta, Georgia 30332, United States; ‡ Centro de Innovación Tecnolóxica en Edificación e Enxeñería Civil (CITEEC), 16737 Universidade da Coruña, A Coruña 15071, Spain

## Abstract

Carbon mineralization pilot projects have demonstrated effective CO_2_ sequestration, yet uncertainties persist for large-scale deployment, particularly regarding the role of fracture networks and evolving fracture-matrix interactions. In this study, we integrate field data, numerical simulations and gravimetric-volumetric analyses to investigate the fracture characteristics of basalt formations and their implications for CO_2_ storage. Fracture aperture is shown to be proportional to block size, governed by thermal contraction during lava cooling, with the aperture-to-block size ratio β ranging from ∼0.7·10^–2^ to 6·10^–2^ depending on mineralogy. Network modeling reveals that initial aperture variability is amplified by dissolution near the injection zone (high Peclet and low Damköhler numbers); however, the hexagonal fracture topology enhances mixing and delays hydrochemical feedback and flow localization. Chemo-gravimetric analysis indicates that mineralization can sequester 0.2–0.3 g CO_2_ per gram of rock, significantly exceeding pore-space storage via supercritical or dissolved CO_2_. However, volume-positive mineralization eventually reduces fracture transmissivity. Aperture shut-off depends on the aperture-to-block size ratio β and the mineralization expansion factor ε. The reacted volume fraction at shutoff can range from 7 to 24%. Mineralogy emerges as a primary control on fracture topology, chemical reactivity and storage capacity. Results underscore the need for careful reassessment of CO_2_ storage capacity in mafic rocks.

## Graphical abstract

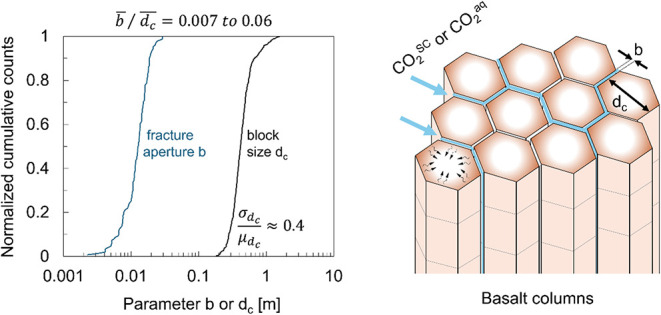

## 1 Introduction

Pilot projects in Iceland (Carbfix^1^) and the United States (Wallula^2^) have shown effective CO_2_ sequestration by carbon mineralization in igneous formations within relatively short time scales.^3^ The injection of a CO_2_-rich aqueous solution into mafic and ultramafic rocks releases divalent metal cations from silicate minerals (e.g., Ca^2+^, Mg^2+^, and Fe^2+^); these cations subsequently react with carbonate ions CO_3_^2–^ to form stable carbonate minerals such as calcite CaCO_3_, magnesite MgCO_3_ and siderite FeCO_3_.^4^ Still, significant uncertainties remain for large-scale deployment.^5–7^

Basalts are primary targets for carbon mineralization because of their mineral composition: iron and magnesium-rich mafic minerals, calcium-rich varieties of plagioclase feldspar, and a relatively low silica content from (∼45–55%). Furthermore, basalts exhibit distinctive fracture networks surrounding quasi-parallel polygonal columns.^8,9^ These high-transmissivity open fractures are typically more permeable than the rock matrix and become preferential flow pathways.^10,11^ Fracture walls provide a reactive surface for dissolution and mineral precipitation.^12^

There are other mafic rocks with compositions similar to that of basalt. Gabbro and diabase have coarser grain sizes because they cooled slowly within intrusive bodies such as dikes and sills.^13,14^ While diabase can exhibit column-bounding fractures (e.g., Ringing Rocks County Park, Pennsylvania), gabbro cools at greater depths beneath the Earth’s surface >5 km and does not develop a systematic fracture network,^15–17^ therefore its low permeability hinders its potential for CO_2_ storage.^18^ Ultramafic peridotite and dunite have higher calcium and magnesium contents than basalts; however, they are typically found in tectonic settings where fracture networks are less developed or at greater depths, which limits accessibility.

Despite the critical role of fractures in reactant transport, current CO_2_ mineralization studies primarily focus on the reactivity of the bulk rock mass, without explicitly considering the role of fracture networks and evolving fracture-matrix interactions. Consequently, a deeper understanding of the fracture network in mafic rocks is necessary to engineer effective injection strategies and to assess the long-term carbon storage potential.^19,20^ In particular, we need to recognize fracture properties such as orientation, spacing, aperture and connectivity, and their effect on ensuing hydro-chemo-mechanical processes.^21–23^

This study advances the understanding of CO_2_ mineralization in fractured basalt formations by focusing on the interplay between fracture topology, reactive transport and storage capacity. The injection of CO_2_ dissolved in water produces an acidic solution that promotes silicate mineral dissolution along fracture surfaces and in the matrix. The released divalent cations such as Ca^2+^, Mg^2+^ and Fe^2+^ subsequently react with carbonate ions to precipitate solid carbonate minerals. Overall, this is a volume-positive reaction. To explore these processes, this work is organized into three main parts: (1) a comprehensive examination of fracture properties by combining data from previous publications and new field data acquired as part of this study; (2) the development of network models -informed by field data and experimental geochemical parameters- to analyze the transport of reactant species and fracture aperture evolution in the near field; and (3) the estimation of CO_2_ storage capacity of naturally fractured basalts in the far field.

## 2 Fracture Formation and Topology – Data Compilation

The formation of columnar structures in basalts starts with crystal nucleation and growth, involves heat diffusion,^24^ and the development of tensile stress during cooling contraction.^25^ Cooling fronts in lava flows migrate from both the top and the bottom boundaries toward the interior of the flow, forming the upper and lower colonnades^26^ (Figure 1A). Colonnades consist of column-bounding parallel-wall fractures that propagate perpendicularly to the cooling front^27^ (Figures 1B and 2A). Thus, fractures are vertical or subvertical in extended flows, and become subhorizontal as lava cools near the lateral edges of the flow^28^ (Figure 2B). Postformation geological stresses such as tectonic forces can modify column orientation. Columns often display a polygonal cross section –e.g., well-developed hexagons– depending on the cooling conditions^29^ (Figure 2C). Lava composition, flow thickness and the presence of water also affect the evolving fracture network within a lava flow.^30,31^

**Figure 1:**
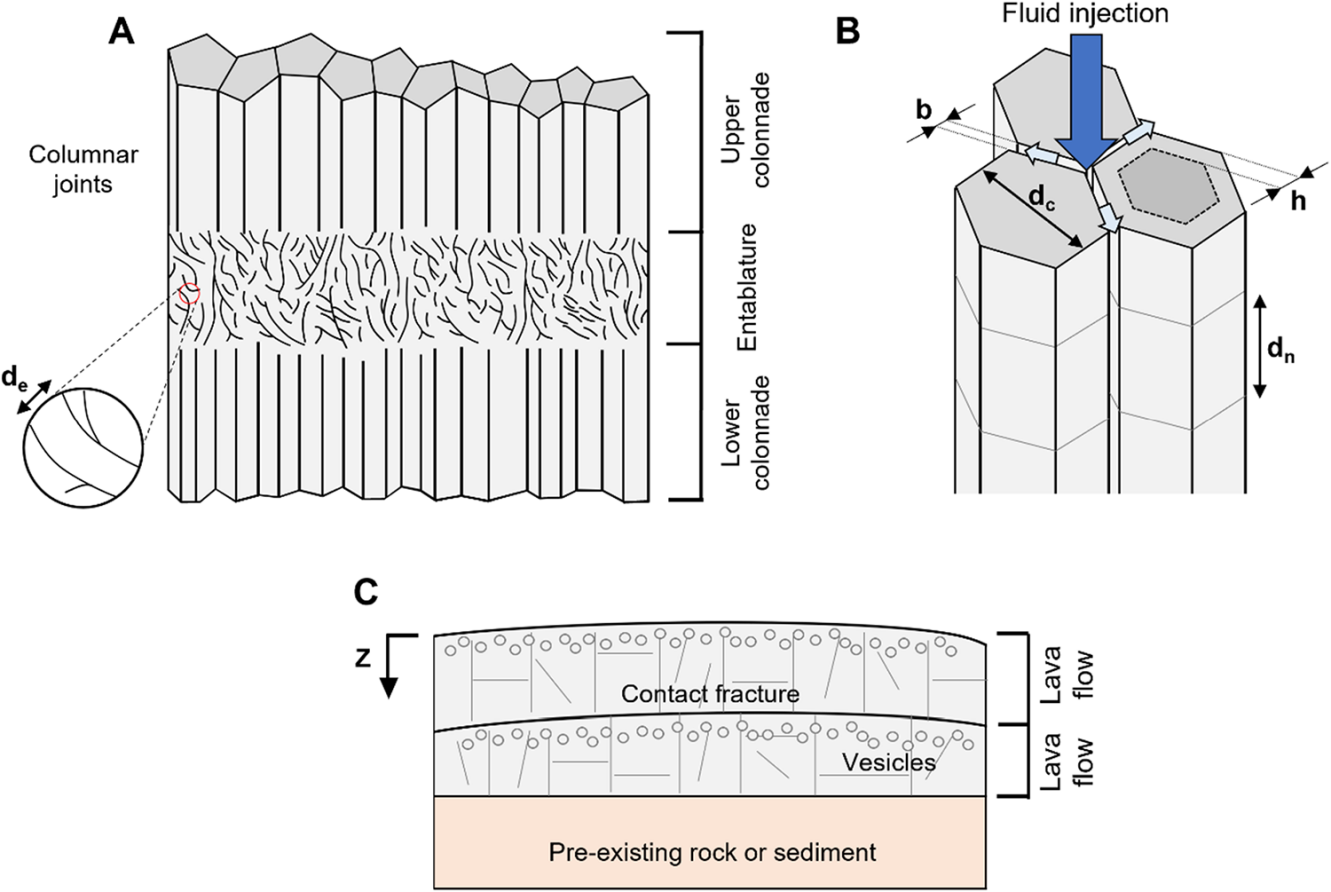
Structure of mafic basalt formations. (A) A basalt lava flow (based on ref 31). (B) Geometry of basalt columns. (C) Lava flows overlying a pre-existing rock or sediment. *Parameters:* *d_e_* = entablature fracture spacing, *d_c_* = block size, *b* = fracture aperture, *d_n_* = column-normal fracture spacing, *h* = reacted thickness, *z* = depth measured from the top of the flow downward.

**Figure 2:**
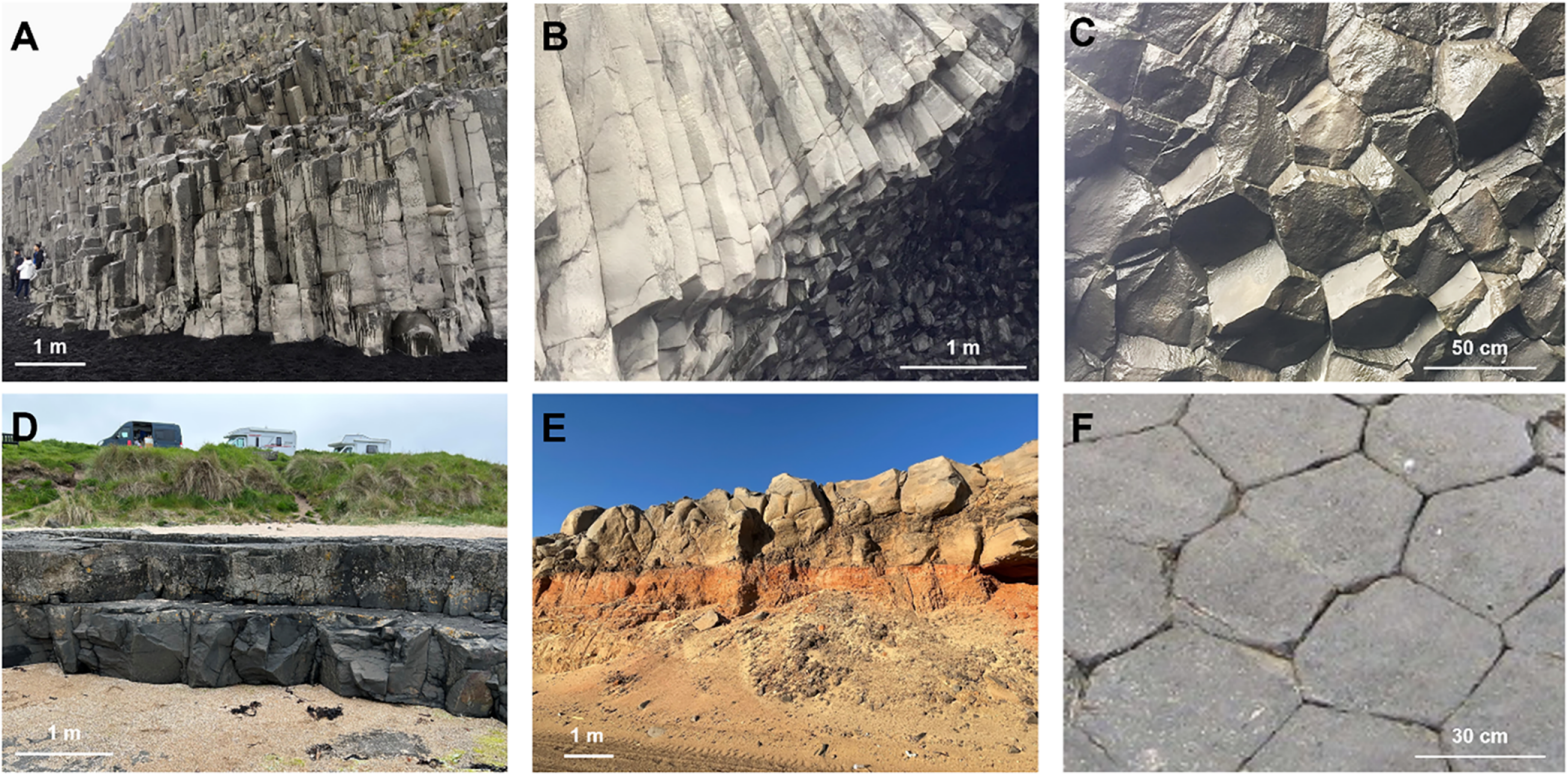
Lava flows and basalt formations. (A–C) Reynisfjara, Iceland—The polygonal cross section shown in picture-C is observed at the base of the columns, as viewed from inside an eroded carven. (D) Berwick-upon-Tweed, England (E) Saudi Arabia. (F) Aubrac, France. Note: scale bars determined from references in the field. (F) is adapted with permission from ref 32. Copyright 2010 Elsevier.

We examine fracture properties in exposed mafic formations using data and photographs reported in previous studies and taken during our visits to various locations in Iceland, Saudi Arabia and England (Table 1).

**Table 1:** Basaltic Formations Analyzed in This Study

formation	location	characteristics
Reynisfjara black beach^a^,^b^,^g^	Iceland	well-defined polygonal columns
Studlagil Canyon^g^	Iceland	vertical columns and entablature
Giant’s causeway^g^	Ireland	extensive and well-pronounced hexagonal columns
Staffa^g^	Scotland	well-developed columns and entablature
Ardtun^g^	Scotland	polygonal columns with varying orientations
Berwick-upon-Tweed^a^	England	contact fractures between thin lava flows with no columnar structure
Garni Gorge^g^	Armenia	distinctive basalt columns
Wadi Zee^a^	Saudi Arabia	fractured vesicular basalt
Harrat Al Fatih^c^	Saudi Arabia	fractured vesicular basalt
Snake River Plain^d^,^e^,^f^	United States	colonnades separated by single entablature
Columbia River Basalt Group^g^	United States	thick lava flows; well-formed hexagonal columns

Sources:.

a Field trips as part of this study.

b Reference 38.

c Reference 39.

d Reference 40.

e Reference 8.

f Reference 9.

g Photos found on the Internet – Multiple sources.

### 2.1 Block Size

The block size *d_c_* is the distance between opposite fractures measured across the column longitudinal axis (Figure 1B). Irregularities in column shape and optical bias can cause inaccuracies, therefore, we took multiple measurements to compensate accidental errors. Observed column size and spacing are similar in outcrops and at depth (deep excavation data from refs 33–36 ). Cumulative distributions for block size, based on 1931 measurements, are plotted in Figure 3A. Formations can be categorized according to block size: small blocks *d_c_* < 0.2 m including Berwick-upon-Tweed (England), Wadi Zee and Harrat Al Fatih (Saudi Arabia); medium-size blocks 0.2 m < *d_c_* < 0.4 m such as in Garni Gorge (Armenia), Reynisfjara Black Beach (Iceland), the Giant’s causeway (Ireland), Ardtun and Staffa (Scotland); and formations with large block sizes *d_c_* > 0.6 m found in the Columbia River Basalt Group and Snake River Plain. The coefficient of variation is the ratio between the mean block size μ­(*d_c_*) and the standard deviation σ­(*d_c_*), and falls withing the range σ/μ ≈ 0.4–0.5 in most cases. Block size follows a log-normal distribution; statistical parameters in logarithmic scale ln­(*d_c_*) can be derived from the mean μ­(*d_c_*) and standard deviation σ­(*d_c_*) computed in linear scale;^37^ in particular, σ_ln(*d_c_*)_ ≈ σ­(*d_c_*)/μ­(*d_c_*) for small coefficients of variation. Accordingly, the slope of the cumulative distributions on Figure 3A reflects the coefficient of variation in block size *d_c_* (see superimposed lines in Figure 3A).

**Figure 3:**
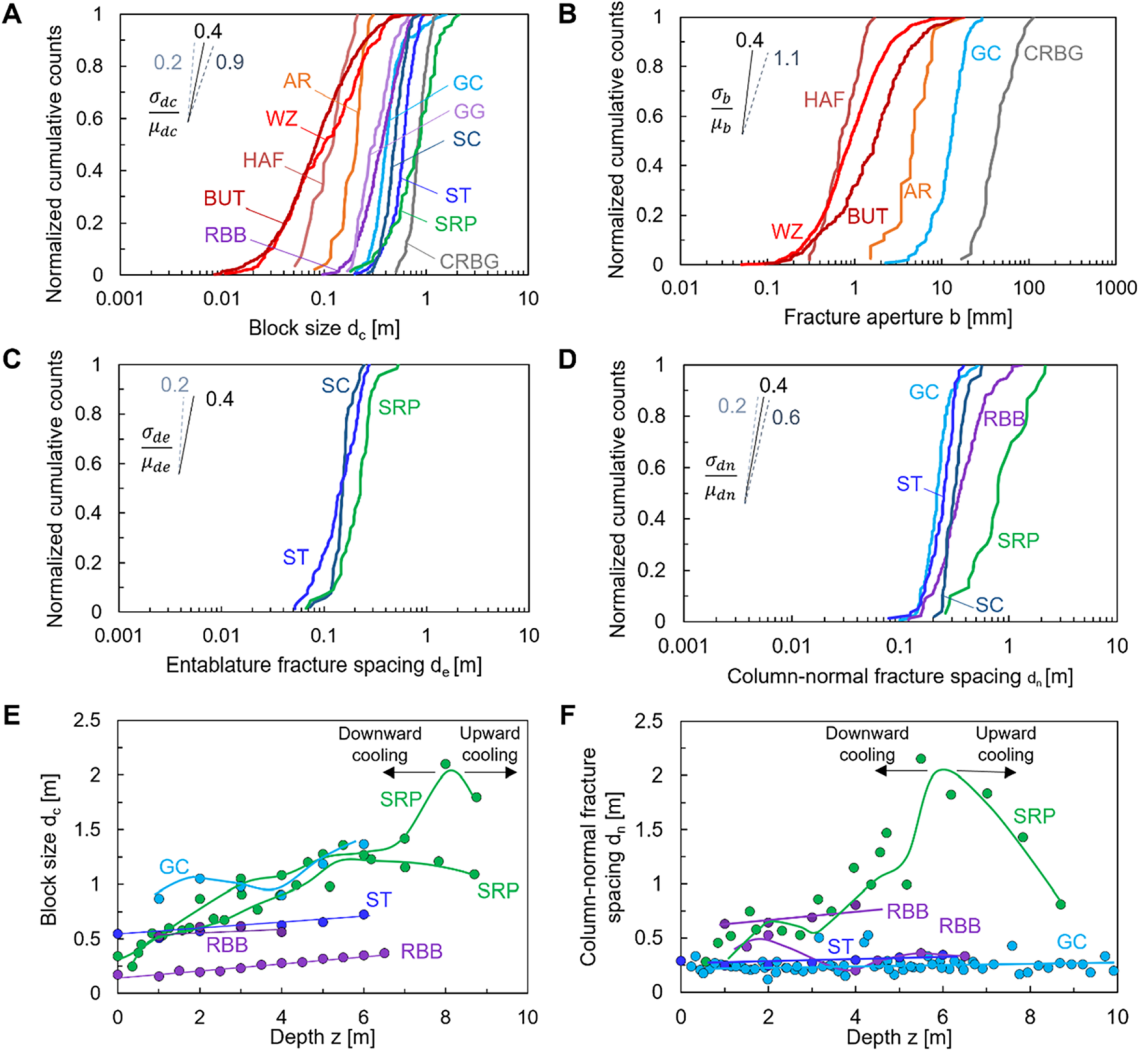
Geometric descriptors of basalt formation — Statistics and trends. (A) Block size *d_c_*. (B) Column-bounding fracture aperture *b*. (C) Entablature fracture spacing *d_e_*. (D) Column-normal fracture spacing *d_n_*. (E) Variation of block size *d_c_* with depth *z*. (F) Variation of column-normal fracture spacing *d_n_* with depth *z*. See Figure 1 for definitions and Table 1 for formation details. Note: AR = Ardtun; BUT = Berwick-upon-Tweed; CRBG = Columbia River Basalt Group; GC = Giant’s Causeway; GG = Garni Gorge; HAF = Harrat Al Fatih; RBB = Reynisfjara Black Beach; SC = Studlagil Canyon; SRP = Snake River Plain; ST = Staffa; WZ = Wadi Zee.

The cooling rate plays a crucial role in determining column size and spacing: faster cooling closer to the surface leads to the formation of smaller columns with narrower gaps between them; column size increases toward the interior of the flow where cooling is slower^8^ (Figure 3E). Furthermore, blocks are typically larger in the lower colonnade which is in direct contact with the ground compared to columns in the upper colonnade which is exposed to the weather and experiences faster cooling.^40^

### 2.2 Fracture Aperture

Aperture determines the fracture transmissivity.^41^ In most cases, fractures are preferential flow pathways unless they are sealed by stress or clogged by gouge and the precipitation of secondary minerals.^42,43^ We measured the fracture geometric aperture *b* as the normal distance between fracture walls observed on exposed surfaces. Image analysis was performed using ImageJ, with a reference element of known size for scale calibration. Multiple measurements were taken along each fracture length to capture aperture variations associated with fracture wall topography. Weathering and optical resolution influence the measured values, with biases having a more pronounced effect on narrower fractures. Nevertheless, the fracture aperture data in Figure 3B reveal valuable trends (2192 values). Fracture apertures vary from *b* < 3 mm (e.g., Harrat Al Fatih, Wadi Zee and Berwick-upon-Tweed) to *b* > 10 mm (e.g., Giant′s Causeway and in the Columbia River Basalt Group). Fracture aperture follows a log-normal distribution, with the standard deviation typically proportional to the mean aperture σ­(*b*)/μ­(*b*) ≈ 0.4 in most data sets; however, some field data show greater variability (once again, the coefficient of variation is the slope of the inclined lines superimposed in Figure 3B; see also ref 44).

Figure 3A,B show that fracture sets with wider mean aperture *b* [m] correspond to formations with larger block size *d_c_* [m]. The proportionality between *b* and *d_c_* can be justified from thermal contraction: consider a thermal expansion coefficient α­[°C^–1^] and a temperature change Δ*T* = *T_f_* – *T*_amb_ [°C] from the fracturing temperature *T_f_* to the ambient temperature *T*_amb_; then:

1 $$b=\alpha \Delta Td_{c}\to \beta =\frac{b}{d_{c}}=\alpha \Delta T$$

The fracturing temperature for basalt ranges between *T_f_* ≈ 840-to-890 °C.^45^ while the thermal expansion coefficient varies from α = 7.63·10^–6^ °C^–1^ to α = 3.90·10^–5^ °C^–1^.^46–48^ Mafic minerals such as pyroxene and olivine exhibit higher thermal expansion coefficients than feldspars^49^). Indeed, the mean aperture *b* and the mean bock size *d_c_* for the various formations explored in this study reveal a mineralogy-dependent ratio β = *b*/*d_c_* between β ≈ 0.7·10^–2^ and β ≈ 6·10^–2^ (Figure 4).

**Figure 4:**
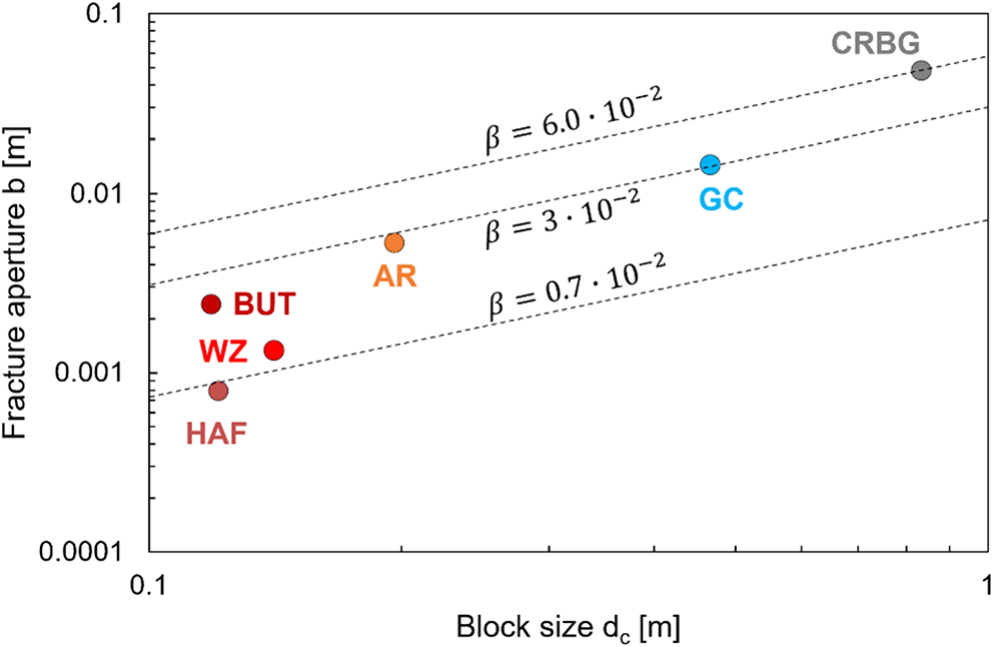
Fracture aperture *b* vs block size *d_c_*. Dashed lines represent various β = αΔ*T* relationships. See Figure 1 for definitions and Table 1 for formation details. Note: AR = Ardtun; BUT = Berwick-upon-Tweed; CRBG = Columbia River Basalt Group; GC = Giant’s Causeway; HAF = Harrat Al Fatih; WZ = Wadi Zee.

### 2.3 Entablature

Zones with irregular fracture structure are called entablature and are characterized by narrow, randomly oriented fractures.^50^ Data gathered for the Snake River Plain (Idaho), Studlagil Canyon (Iceland) and Staffa (Scotland) formations show that block size is smaller in entablatures than in colonnades (Figure 3C—195 values; the coefficient of variation is the slope of the inclined lines superimposed in Figure 3C). The presence of dendritic oxides and glassy interstitial material between the primary minerals observed in the entablature, but absent in the colonnade, and striae propagation directions confirm faster cooling driven by sporadic water invasion.^51^ In fact, dry and wet periods can result in alternating entablature and colonnade layers, with abrupt transitions between them.^31,52^

### 2.4 Column-Normal Fractures

Basalt columns have regularly spaced transverse fractures (Figure 2B). Their spacing *d_n_* scales with the column width *d_c_*, as reflected in the compiled database (Figure 3D — 491 values; the coefficient of variation is the slope of the inclined lines, see also ref 45). Transverse fractures form when the tips of the column-bounding fractures approach the glass transition isotherm and lava cannot flow toward the cooling and contracting column core; then, internal tension develops in the core against the cooler and stiffer column periphery (see also ref 53). In addition, trapped gas bubbles in the cooling lava cause stress concentration and become nucleation sites for the formation of normal fractures (Figure 1C; see also ref 9).

### 2.5 Interlayer Contact Fractures

Vertically stacked sequential lava flows develop interlayer contact fractures from the interplay of thermal contraction and mechanical stresses caused by hot lava flowing and contracting above the pre-existing layer^52,54^ (Figures 1C and 2D). The molten lava alters the underlying rock or soil–paleosols or “red earths”–leading to the creation of distinct layers with varying mineral composition and textures^55,56^ (Figure 2E).

### 2.6 Postformation Alterations

While older lava flows sit deeper in a stack, weathering reflects not only age but also environmental conditions and the exposure time between sequential flows. Thus, deeper layers may have been protected by the overlying younger layers and experienced less weathering.^57,58^ In addition, other postemplacement alterations can modify the fracture network such as crosscutting faults in volcanic rift zones.^59^ Contrary to columns, tectonic faults are not restricted to a single flow and evolve as column-bounding fractures coalesce.^42,60,61^

## 3 Implications and Discussion

### 3.1 Concurrent Processes – Dominant Effects

The injected fluid infiltrates the matrix when the internal matrix porosity forms percolating pathways.^62^ For flow normal to the column length, the ratio between the flow rate through the fractures *q_f_* [m^3^/s] and the matrix *q_m_* [m^3^/s] is related to the transmissivity in the fractures τ [m^2^/s] = γ*b* ^3^/12μ_*d*_ ^22^ and the matrix hydraulic conductivity *K* [m/s]:

2 $$\frac{q_{f}}{q_{m}}=\frac{\tau \mathrm{iH}}{Kd_{c}\mathrm{iH}}=\frac{\gamma b^{3}}{12\mu_{d}Kd_{c}}=\frac{\gamma }{12\mu_{d}K}\beta^{3}d_{c}^{2}$$

The hydraulic gradient *i* [m/m] and the column length *H* [m] cancel out, and the flow ratio becomes a function of the geometric parameters β and *d_c_*, and the matrix conductivity. For typical basalt values (β ≈ 3·10^–2^, *d_c_* ≈ 0.3 m, and *K* ≈ 10^–7^ m/s^63^) and water parameters (γ = 10 kN/m^3^, μ*_d_* = 8.9·10^–7^ kPa·s), the anticipated flow ratio is *q_f_*/*q_m_* ≫1; hence, we anticipate fracture-dominant flow in most fractured basalts. Preferential flow through fractures will be accentuated away from the lava cooling boundaries as wider fracture apertures b developed at lower cooling rates. Equation 2 underscores the role of mineralogical composition captured in the proportionality factor β = *b*/*d_c_* = αΔ*T* (eq 1).

The interplay between advection, diffusion and reactivity controls the spatiotemporal evolution of dissolution and precipitation during carbon mineralization. In a rock mass with fracture-dominant flow conditions *q_f_*/*q_m_* ≫1, the time scales are associated with fluid transport along fractures *t*_adv_, diffusion into the matrix *t*_diff_ and chemical reactions *t*_react_. Consider the flow velocity in fractures *v* [m/s], the column characteristic width *d_c_* [m], the fracture aperture *b* [m], the diffusion coefficient in the matrix *D* [m^2^/s] and the rate constant κ [m/s]; then, the characteristic time scales are:

$$advection\,\;\,\;\,along\,\;\,\;fractures\,\;\;\;\;t_{adv}=\frac{d_{c}}{v}=\frac{d_{c}}{\frac{q_{f}}{bH}}=\frac{d_{c}\mathrm{bH}}{q_{f}}$$ 3

$$diffusion\,\;\,\mathrm{int}o\,\;the\,\;\,matrix\,\;\;\;\;\;t_{\mathrm{diff}}=\frac{{\left(\frac{d_{c}}{2}\right)}^{2}}{D}=\frac{{d_{c}}^{2}}{4D}$$ 4

$$reaction\,\;\;\;t_{\mathrm{react}}=\frac{d_{c}}{2\kappa }$$ 5

Adopting typical parameters for fractured basalts identified in this study (e.g., *d_c_* ≈ 0.3 ± 0.12 m, β = 3·10^–2^ and *b* = β·*d_c_* ≈ 9 ± 3.6 mm), a diffusion coefficient *D* = 2·10^–9^ m^2^/s,^64^ a fracture flow rate per unit fracture height in the near field *q_f_*/*H* ≈ 2.5·10^–3^-to-2.5·10^–4^ m^2^/s (computed considering a target storage formation thickness *H* ≈ 400 m and an injection rate ∼10-to-100 kg/s;^65,66^ and a rate constant κ ≈ 10^–10^ m/s, the estimated times are *t*_adv_ ≈ 10^2^-to-10^3^ s, *t*_diff_ ≈ 10^6^-to-10^7^ s and *t*_react_ ≈ 10^10^ s. Clearly, advection through the fractures is much faster than diffusion into the matrix or chemical reactions.

These time scales can be combined into two dimensionless time ratios, Péclet’s number *Pe* and Damkhöler’s number *Da*:

6 $$\mathrm{Pe}=\frac{t_{\mathrm{diff}}}{t_{\mathrm{adv}}}=\frac{\gamma i\beta^{2}d_{c}^{3}}{48\mu_{d}D}\gg 1$$

7 $$\mathrm{Da}=\frac{t_{\mathrm{adv}}}{t_{\mathrm{react}}}=\frac{24\mu_{d}\kappa }{\gamma ib^{2}}\ll 1$$

These ratios define the regimes for governing processes in the two-dimensional *Pe*–*Da* space;^67–69^ for fractures, see ref 70. For *Pe* ≫ 1 and *Da* ≪ 1, we can anticipate a ramified dissolution pattern along dominant interconnected fractures.

### 3.2 Dissolution near the Injection Well – Network Model

Acidic, CO_2_-charged water near the injection well favors the formation of bicarbonate ions HCO_3_^–^; additionally, large flow rates prevent the accumulation of reaction products until some radial distance from the injection point.^71^ Therefore, dissolution prevails near the injection well.

We ran fracture network simulations to explore the effects of dissolution in a fractured mafic formation. All analyses are normalized by the column’s height *H*, thus, the three-dimensional (3D) columnar structure reduces to a two-dimensional hexagonal fracture network, with null volume at nodes. When combined with the dominant influence of aperture size distribution, this simplified network topology enables robust analysis of the coupled hydro-chemical processes occurring during CO_2_ geological storage.

The fractures that define the regular hexagonal columns have an aperture *b* [m] and length *L* = **d*_c_*/√3. The flow rate per unit fracture height *q* [(m^3^/s)/m] satisfies the cubic law and is a function of the fluid dynamic viscosity μ*_d_* [Pa·s] and pressure gradient Δ*P*/*L* [Pa/m],

8 $$q=\frac{b^{3}\Delta P}{12\mu_{d}L}$$

The sum of all flow rates in-and-out of the *u*-node is zero (mass conservation):

9 $$\sum q_{i}=0=\frac{1}{12\mu_{d}L}\sum_{u^{*}}b_{i}^{3}\left(P_{\mathrm{nu}}-P_{\mathrm{nu}*}\right)$$

where the subscripts refer to the *i*-fractures that link neighboring u*-nodes to the *u*-node.

The H^+^-concentration in the *i*-fracture decreases with time as the fluid reacts with the rock surface. Let us assume first-order reaction kinetics so that the fluid-mineral reaction rate *R* [mol_rock_/(m^2^s)] = κ*C*_*fi*_δ is proportional to the H^+^-concentration *C*_*fi*_ [mol_H^+^_/m^3^] in the fluid, the rate constant κ [m/s] and the stochiometric factor δ [mol_rock_/mol_H^+^_] that relates rock dissolution to consumed H^+^. This is also the rate of reactant loss in the fluid during the time interval Δ*t* [s]; then the updated concentration of reactants in the fracture is

10 $$C_{\mathrm{fi}}^{j}=C_{\mathrm{fi}}^{j-1}-\frac{R\left(2A_{f}\right)}{V_{f}}\Delta t=C_{\mathrm{fi}}^{j-1}-\frac{2}{b_{i}^{j-1}}\kappa C_{\mathrm{fi}}^{j-1}\delta \Delta t$$

where the ratio between the fracture volume to its area is the fracture aperture *b* [m] = *V_f_*/*A_f_*, and the factor 2 reflects the contribution of the two walls. We focus on fracture surface dissolution and disregard diffusive transport into the matrix and internal reactivity. This assumption underestimates the rate of H^+^ consumption and, therefore, the overall reaction rate; however, it is a good approximation near the injection well where the advection time along fractures is much shorter than the diffusion time into the matrix (eq 6).

The fracture H^+^-concentration *C*_*fi*_^*j*^ reaches the outlet *u*-node at time *t_j_*. If more than one fracture feeds the *u*-node, then the node concentration *C*_*nu*_^*j*^ is the volume average of the concentrations *C*_*fk*_^*j*^ in the *k*-feeding fractures (assuming instantaneous mixing):

11 $$C_{\mathrm{nu}}^{j}=\frac{\sum_{k}q_{k}^{j}C_{\mathrm{fk}}^{j}}{\sum_{k}q_{k}^{j}}$$

The concentration *C*_*nu*_^*j*^ [mol/m^3^] at the *u*-node becomes the inlet concentration for fractures that feed from the *u*-node.

The fluid coming out of the u-node displaces the fluid in the fracture to a distance proportional to the time step Δ*t* relative to the residence time *t*_*r*_ [s] = *Lb*/*q*. Then, the mean H^+^-concentration in the *i*-fracture *C*_*fi*_^*j*^ is a weighted combination of the concentration at the inlet *u*-node *C*_ *nu*_^*j*–1^ [mol/m^3^] and the current fracture concentration *C*_*fi*_^*j*–1^ [mol/m^3^] according to Δ*t*/*t*_*r*_:

12 $$C_{\mathrm{fi}}^{j}=C_{\mathrm{nu}}^{j-1}\frac{\Delta t}{t_{r}}+C_{\mathrm{fi}}^{j-1}\left(1-\frac{\Delta t}{t_{r}}\right),\mathrm{for}\;{\Delta t\leq t}_{r}\left[s\right]$$

The change in H^+^-concentration Δ*C*_*fi*_^*j*^ [mol/m^3^] in the fracture fluid produces a change in fracture aperture Δ*b* [m]:

13 $$\Delta b_{i}^{j}\mathrm{HL}=\left[q_{i}^{j}\Delta t\left(C_{\mathrm{fi}}^{j}-C_{\mathrm{fi}}^{j-1}\right)\right]\lambda \to \Delta b_{i}^{j}=\frac{\left[q_{i}^{j}\Delta t\left(C_{\mathrm{fi}}^{j}-C_{\mathrm{fi}}^{j-1}\right)\right]\lambda }{\mathrm{HL}}$$

where *q*/*H* is the flow per unit column heigh. The updated fracture aperture *b*_*i*_^*j*^ [m] becomes

14 $$b_{i}^{j}=b_{i}^{j-1}+\Delta b_{i}^{j}$$

The factor λ [m_^3^rock_/mol_H^+^_] in eq 13 relates the volume of rock dissolved per mol of protons H^+^ consumed. The stochiometric dissolution of basaltic glass is^72,73^

15 $$\mathrm{Si}{\mathrm{Al}}_{0.356}{\mathrm{Fe}}_{0.190}{\mathrm{Mg}}_{0.281}{\mathrm{Ca}}_{0.264}{\mathrm{Na}}_{0.079}K_{0.008}O_{3.315}+2.630H^{+}+0.685H_{2}O=H_{4}\mathrm{Si}O_{4}+0.358{\mathrm{Al}}^{3+}+0.190{\mathrm{Fe}}^{2+}+0.281{\mathrm{Mg}}^{2+}+0.264{\mathrm{Ca}}^{2+}+0.079{\mathrm{Na}}^{+}+0.008K^{+}$$

Consequently, one mol of H^+^ dissolves 1/2.63 mol of glass, hence, the stochiometric factor in eq 10 is δ = 1/2.63 [mol_rock_/mol_H^+^_]. As the molar volume of glass *V_m_* = 4.2·10^–5^ m^3^_rock_/mol_rock_, the factor λ [m^3^_rock_/mol_H^+^_] is

16 $$\lambda =\delta V_{m}=\frac{1{\mathrm{mol}|}_{\mathrm{rock}}}{{2.63\mathrm{mol}|}_{H^{+}}}4.2\cdot 10^{-5}\frac{{m^{3}|}_{\mathrm{rock}}}{{\mathrm{mol}|}_{\mathrm{rock}}}=1.6\cdot 10^{-5}\frac{{m^{3}|}_{\mathrm{rock}}}{{\mathrm{mol}|}_{H^{+}}}$$

Similar results are obtained using the mineralogical compositions of basaltic formations from other locations, including the United States, China, New Zealand and Austria.^74–78^ The resulting values span δ ≈ 1/3.5-to-1/2.3 mol_rock_/mol_H^+^_, *V*_m_ ≈ 4.1·10^–5^-to-4.8·10^–5^ m_^3^rock_/mol_rock_ and λ ≈ 1.4·10^–5^-to-1.8·10^–5^ m_^3^rock_/mol_H^+^_.

These equations are implemented in a MATLAB algorithm. Initially, the reservoir is saturated with water in equilibrium with the formation (pH = 8.5; refs 79,80 ). A constant-pressure boundary condition is set at the outer edge of the domain, located approximately ten times the block size ∼10*d_c_* away from the injection point. Then, water with dissolved CO_2_ is injected at the center (for pressure and temperature conditions similar to the Carbfix pilot project: pH = 3.2 -concentration of H^+^ = 6.31·10^–4^ mol/L;^81^). As reactive water invades the network, hydro-chemical conditions are gradually updated as follows: (1) simultaneously solve the instantaneous nodal pressures *P_nu_* throughout the network to satisfy mass balance at each node (eq 9); (2) compute flow rate *q* (eq 8) and flow velocity *v* = q/b for each fracture; (3) determine the mean H^+^-concentration in the fractures (eq 12); (4) compute the changes in concentration (eq 10); (5) update fracture apertures (eqs 13 and 14); (6) update nodal concentrations at all nodes (eq 11).

For this study, simulation conditions focused on fracture aperture variability (simulation parameters are listed in Table 2). Figure 5 shows the initial fracture aperture distribution, the normalized change in fracture aperture, the spatial distribution of excess H^+^ and the flow rate *q* for two cases:

**Figure 5:**
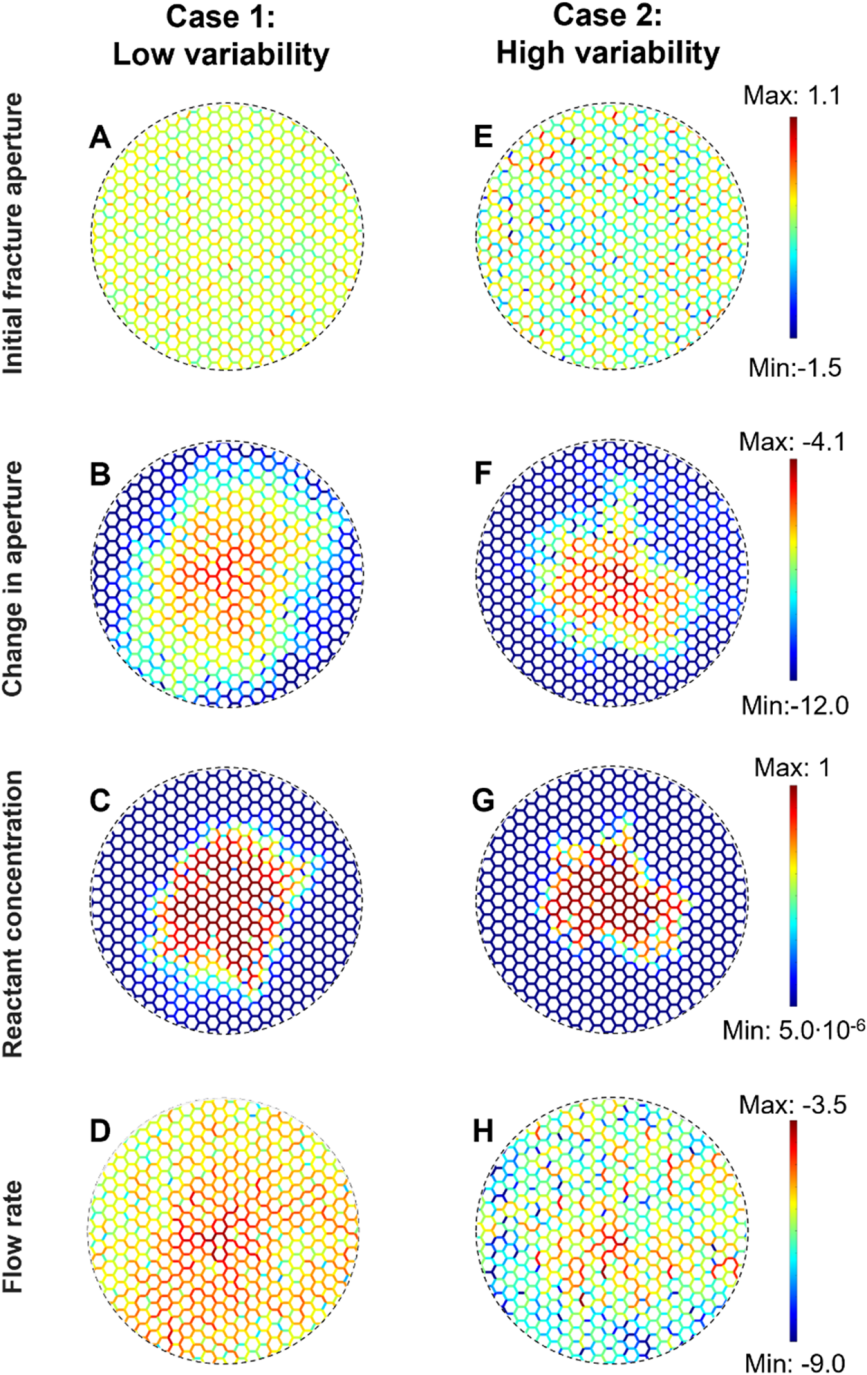
Reactive flow in the near field: Dissolution – Hexagonal fracture network model with constant flow rate injection at the center. Left column: low aperture variability μ­(b) = 1 mm and σ/μ = 0.4. Right column: high aperture variability μ­(*b*) = 1 mm and σ/μ = 1.0. (A&E) Initial fracture aperture – color scheme linear with log­(b/mm). (B&F) Normalized change in fracture aperture at the end of injection – color scheme linear with log­(Δ*b*/*b*_0_). (C&G) Reactant concentration at the end of injection normalized by the inlet concentration – color scheme linear with [H^+^]/[H^+^]_inlet_. (D&H) Fluid flow rate – color scheme linear with log­(*q*/(m/s)). Note: Logarithmic scales are used to highlight differences and capture variations across several orders of magnitude. See network model characteristics and simulation parameters in Table 2.

**Table 2:** Network Model Characteristics and Simulation Parameters

**Parameter**	**Value**
Mineralogy	Basaltic glass
Rock molar mass *m*_R_ [g/mol]^a^	120.9
Rock molar volume *V*_R_ [m^3^/mol]	4.2·10^–5^
Rock density ρ_R_ [kg/m^3^]	2900
Network size	50 × 48
Injection fluid pH [ ]^b^	3.2
Injection fluid H^+^ concentration [mol/L]	6.31·10^–4^
Initial reservoir fluid pH [ ]^c^	8.5
Initial H^+^ concentration in the reservoir [mol/L]	3.16·10^–9^
Rate constant κ [m/s]	1.65·10^–10^
Mean fracture aperture μ(*b*) [mm]	1
Block size *b_c_* [m]	0.3
Fluid dynamic viscosity μ*_d_* [Pa s]	8.9·10^–4^

Sources:

a Computed based on the chemical composition of basaltic glass defined in eq 15;^72,73^

b Reference 69.

c References 79,80 .

*Case 1*. *Low aperture variability* (Figure 5A: log-normal, μ­[mm] = 1, σ/μ = 0.4). There are minor changes in aperture near the injection well (Figure 5B) where reactants are more readily consumed (Figure 5C). The hexagonal fracture network imprints its topology on flow patterns even though there is a random aperture field (Figure 5D).

*Case 2. Large aperture variability* (Figure 5E: log-normal, μ­[mm] = 1, σ/μ = 1.0). Interconnected wider fractures transport reactants more efficiently, resulting in higher dissolution rates that further enlarge initial aperture differences (Figure 5F,G).

The cubic law of fracture transmissivity τ ∝ *b* ^3^ amplifies aperture variability and can cause fluid flow channeling along a small fraction of fractures.^82^ For example, less than <10% of fractures in a parallel fracture set carry 90% of the flow when the aperture variability σ/μ ≈ 0.4;^44^ this is confirmed by analogous simulations conducted using a single set of parallel fractures (Figure 6—Note: The algorithm can be readily applied to any fracture network topology, as demonstrated in this case). However, results in Figure 5 show that the hexagonal fracture topology mixes flow at every node/intersection and delays flow channeling; for example, 10% of the quasi-radial fractures carry ∼40% of the flow in a hexagonal fracture network for the same aperture variability σ­(b)/μ­(b) = 0.4 (Figure 5D—See also^44,82^ ). Still, dissolution in the nearfield amplifies initial differences in transmissivity and can lead to flow localization along high-transmissivity preferential paths in fractured basalts.

**Figure 6:**
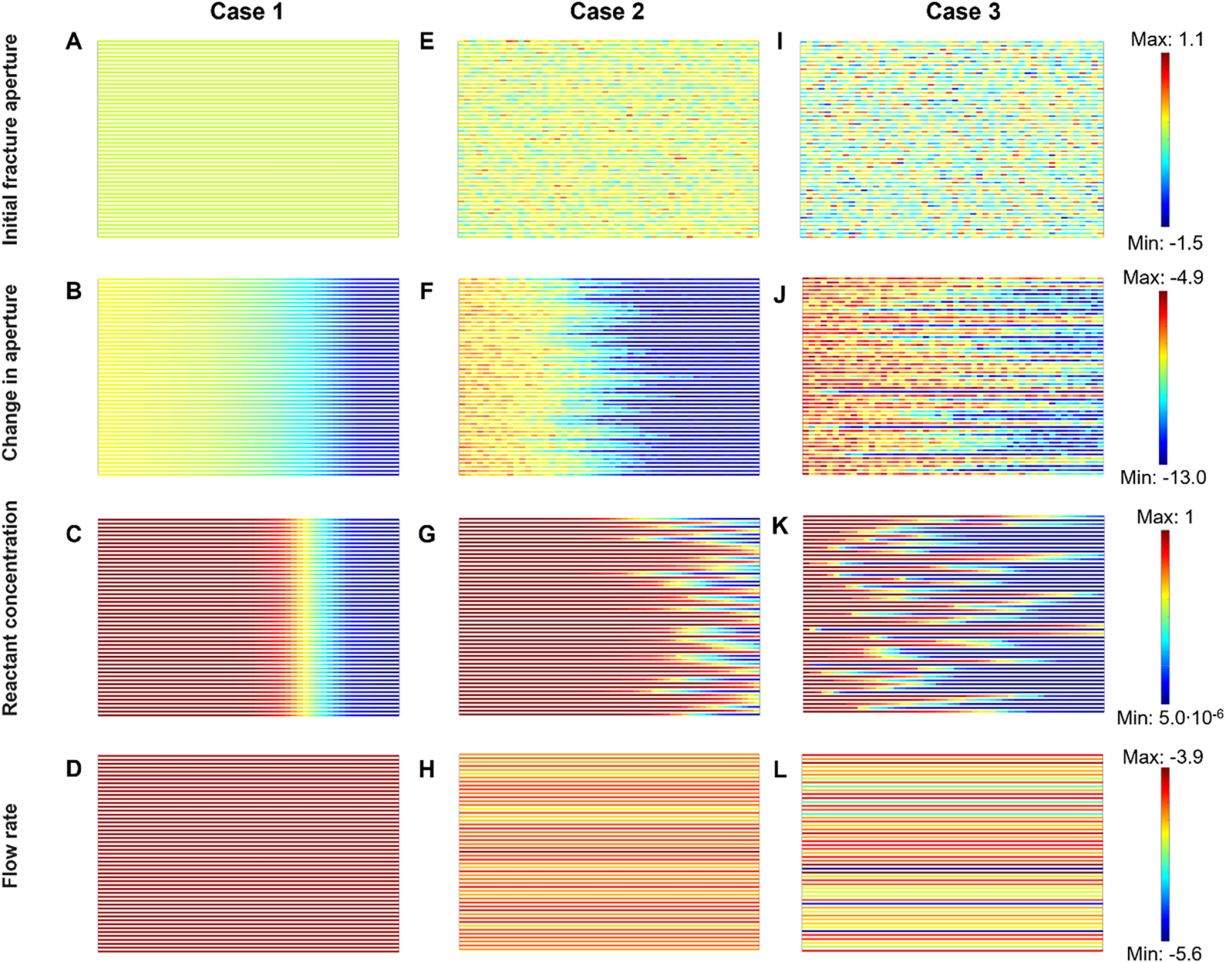
Reactive flow and dissolution along parallel fractures subjected to constant inlet and outlet pressures. Left column: no initial aperture variability μ­(*b*) = 1 mm and σ/μ = 0. Central column: low aperture variability μ­(*b*) = 1 mm and σ/μ = 0.4. Right column: high aperture variability μ­(*b*) = 1 mm and σ/μ = 1.0. (A, E, I) Initial fracture aperture – color scheme linear with log­(*b*/mm). (B, F, J) Normalized change in fracture aperture at the end of injection – color scheme linear with log­(Δ*b*/*b*_0_). (C, G, K) Reactant concentration at the end of injection normalized by the inlet concentration – color scheme linear with [H^+^]/[H^+^]_inlet_. (D, H, L) Fluid flow rate – color scheme linear with log­(*q*/(m/s)). The is increased flow channeling as the fracture aperture variability increases. Note: Logarithmic scales are used to highlight differences and capture variations across several orders of magnitude. See network model characteristics and simulation parameters in Table 2.

### 3.3 Mineralization in the Far Field – CO_2_ Storage Capacity

Reaction products accumulate and pH gradually increases away from the injection point until precipitation conditions are reached.^62^ Precipitation reduces transmissivity and flow diverts to alternative pathways; therefore, contrary to dissolution, precipitation is self-homogenizing, hence, evenly distributed precipitation is expected away from the injection well.

Building on the fracture topology developed in Section 2, we can assess the contribution of fractures to CO_2_ storage capacity. The total representative column volume per unit column height *V_c_^T^* [m^3^/m] is defined between nodal centers at fracture intersections (see Figure 7); for hexagonal columns with constant fracture spacing *d*_c_ and aperture *b*,

$$representative\,\;volume\,\;\;\;\;V_{c}^{T}=\frac{\sqrt{3}}{2}{\left(d_{c}+b\right)}^{2}$$ 17

**Figure 7:**
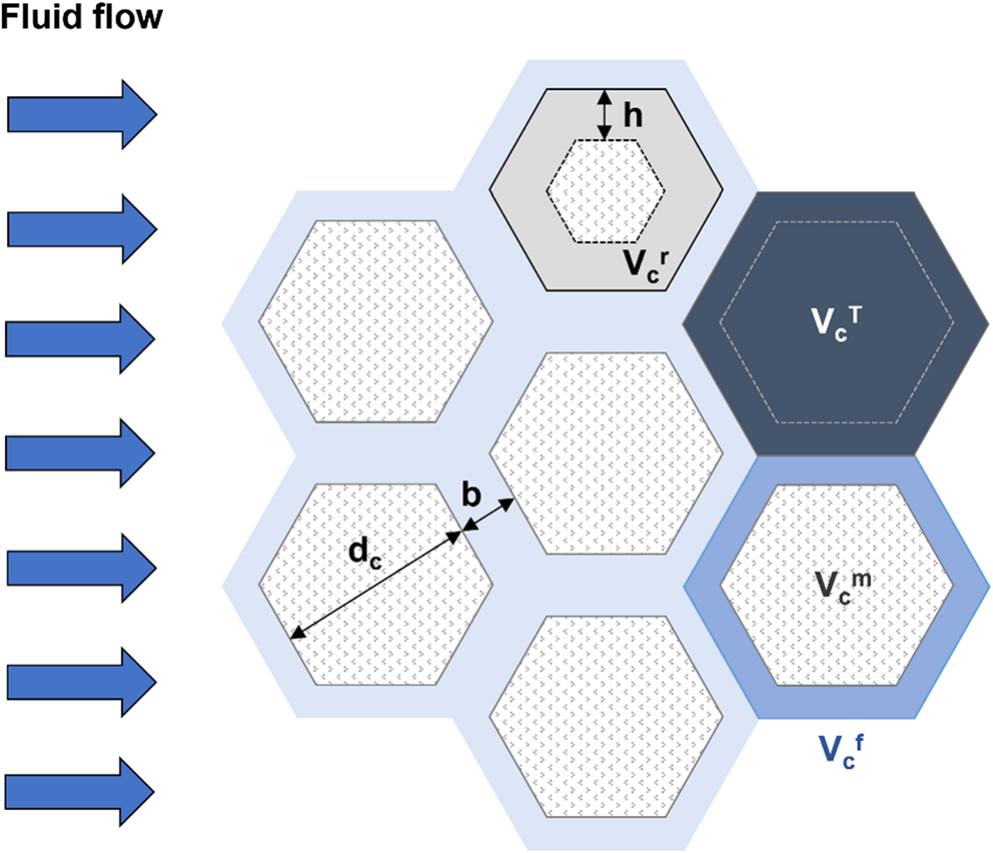
Storage capacity – Definition of the various volumes per column: total representative volume *V_c_^T^*, matrix volume *V_c_^m^*, fracture volume *V_c_^f^* and the reactive volume *V_c_^r^*. Geometric parameters: *b* = fracture aperture, *d*_c_ = block size, *h* = peripheral layer thickness involved in reactions.

If a layer of thickness *h* [m] is involved in chemical reactions around the column perimeter, the reactive volume *V_c_^r^* [m^3^/m] per unit column height is

$$reactive\,\;\;volume\,\;\;\;\;\;V_{c}^{r}=\frac{\sqrt{3}}{2}d_{c}^{2}-\frac{\sqrt{3}}{2}{\left(d_{c}-2h\right)}^{2}=2\sqrt{3}\left(d_{c}h-h^{2}\right)$$ 18

The rock mass that undergoes mineralization per unit column height M_R_ [kg/m] = *V_c_^r^* ρ_R_ is defined by the reactive volume *V_c_^r^* and the rock bulk density ρ_R_ [kg/m^3^].

The rock′s initial mineralogy dictates the reactions that transform the original rock composition into secondary minerals, and the mass of CO_2_ stored in carbonate minerals *M*_CO2,*i*_ [kg].^83^ Let′s assume that all the Ca^2+^, Mg^2+^ and Fe^2+^ ions locally released during rock dissolution are fully converted at the same location into carbonate minerals calcite CaCO_3_, magnesite CaCO_3_ and siderite FeCO_3_. The mass of CO_2_ trapped per initial mass of rock *M*_CO2_/M_R_ takes into consideration the quantity of each cation in the original rock ψ_i_ [mol_cation_/mol_rock_], the amount of carbonate that can be formed with each cation φ_i_ [mol_carbonate_/mol_cation_], the amount of CO_2_ trapped in each carbonate θ_i_ [mol_CO2_/mol_carbonate_] and the molar mass of carbon dioxide *m*_CO_2__ = 44 gr_CO2_/mol_CO_2__. Then, the mass of mineralized CO_2_ relative to the initial rock mass is

19 $$\frac{M_{\mathrm{CO}2}}{M_{R}}\left[\frac{{\mathrm{gr}|}_{\mathrm{CO}2}}{{\mathrm{gr}|}_{\mathrm{rock}}}\right]=\frac{m_{\mathrm{min CO}2}\left[\frac{{\mathrm{gr}|}_{\mathrm{CO}2}}{{\mathrm{mol}|}_{\mathrm{rock}}}\right]}{m_{R}\left[\frac{{\mathrm{gr}|}_{\mathrm{rock}}}{{\mathrm{mol}|}_{\mathrm{rock}}}\right]}=\frac{\sum_{i}\psi_{i}\varphi_{i}\theta_{i}m_{\mathrm{CO}2}}{m_{R}\left[\frac{{\mathrm{gr}|}_{r}}{{\mathrm{mol}|}_{r}}\right]}$$

Consider basaltic glass as in eq 15. Its composition *Si* + *Al*_0.356_ + *Fe*_0.190_ + *Mg*_0.281_ + *Ca*_0.264_ + *Na*_0.079_ + *K*_0.008_ + *O*_3.315_ determines its molar mass m_R_ = 120.9 g/mol, and the quantity of each cation in the original rock: ψ_Ca_ = 0.264, ψ_Mg_ = 0.281, and ψ_Fe_ = 0.190. Similarly, the values φ_i_ and θ_i_ are determined from the corresponding stoichiometric relations and are equal to 1.0 for the three carbonate minerals considered. Finally, the ratio between the mass of mineralized CO_2_ relative to the initial rock mass is

20 $$\frac{M_{\mathrm{CO}2}}{M_{R}}=0.267\frac{{\mathrm{gr}|}_{\mathrm{CO}2}}{{\mathrm{gr}|}_{\mathrm{rock}}}$$

We incorporate a reduction coefficient η_min_ [ ] to account for the reduced efficiency of CO_2_ mineralization due to the formation of other secondary minerals such as clay and zeolites (η_min_∼ 0.25-to-0.4 in 2 years, which represents the fraction of carbonates precipitated relative to the total minerals formed;^84,85^ ) and relate the rock mass to its volume using the rock bulk density ρ_R_ [kg/m^3^] to take porosity into consideration. Then, the mass storage capacity per representative column volume *M*_CO_2__/*V_c_^T^* [kg/m^3^] is

21 $$\frac{M_{CO_{2}}}{V_{c}^{T}}=\frac{V_{c}^{r}}{V_{c}^{T}}\rho_{R}\eta_{\min}\left(\frac{M_{\mathrm{CO}2}}{M_{R}}\right)=\frac{{4\left[\frac{h}{d_{c}}-\left(\frac{h}{d_{c}}\right)\right]}^{2}}{{\left(1+\beta \right)}^{2}}\rho_{R}\eta_{\min}\left(\frac{M_{\mathrm{CO}2}}{M_{R}}\right)\mathrm{for}\;\,h\leq \mathrm{dc}/2$$

How far does carbonization extend into the rock matrix, in other words, is there a limit for the reactive layer thickness *h*? Carbon mineralization is a volume-positive reaction, with volumetric strain ε = Δ*V*/*V* ≈ 0.2 for peridotite and ε ≈ 0.4-to-0.6 for mafic silicate minerals, including olivine and its magnesium-rich end-member forsterite.^86–89^ The resulting expansion can eventually close fractures and shut off fluid flow.^90^ Then, the thickness h [m] of the peripheral zone that undergoes carbonation is ultimately constrained by the fracture self-sealing (Note: some carbonization into the matrix porosity takes place beyond the thickness *h*):

22 $$\varepsilon \cdot h=\frac{b}{2}\to h=\frac{b}{2\varepsilon }$$

Then, there is partial mineralization at shutoff and the storage capacity becomes (refer to eq 21):

23 $${\frac{M_{CO_{2}}}{V_{c}^{T}}|}_{@\mathrm{shutoff}}\approx \frac{2\beta }{\varepsilon }\rho_{R}\eta_{\min}\left(\frac{M_{\mathrm{CO}2}}{M_{R}}\right)$$

where the approximation applies for β = *b*/*d*_c_ ≪ 1 (eq 1 and Figure 4). The column volume per unit height *V_c_^m^* [m^3^/m] is:

$$column\,\;\,volume\,\;\;\;\;V_{c}^{m}=\frac{\sqrt{3}}{2}d_{c}^{2}$$ 24

Then, the ratio between the reactive volume *V_c_^r^* at shut off (eq 18) and the column volume is:

25 $$\frac{V_{c}^{r}}{V_{c}^{m}}=4\frac{\left(d_{c}h-h^{2}\right)}{d_{c}^{2}}=4\left(\frac{\beta }{2\varepsilon }-\frac{\beta^{2}}{4\varepsilon^{2}}\right)\approx \frac{2\beta }{\varepsilon }$$

where the approximation assumes small β. Combining representative values based on mineralogical context, the reactive volume ranges between *V_c_^r^*/*V_c_^m^* ∼7% and 24% of the column volume (Considers: ε ≈0.2 for peridotite formations with low aperture-to-block size ratios such as those in Saudi Arabia, and ε ≈ 0.5 for olivine-rich systems such as the Giant′s Causeway or the Columbia River Basalt Group—see Figure 4). In a multimineral system, differential stress develops within the rock mass due to spatial heterogeneity, variations in reaction kinetics and associated volume changes. Over time, the evolving stress field promotes localized degradation. Subsequently, initially unstressed precipitates gradually begin to exert swelling pressures as fracture shutoff progresses.

Table 3 compares the anticipated CO_2_ storage capacity via mineralization under two scenarios: full matrix reaction and at fracture shut-off caused by volume-positive reactions. Fracture shut-off significantly reduces the mineralization potential. The results emphasize that formations with a high aperture ratio β = *b*/*d_c_* offer more favorable conditions for CO_2_ storage.

**Table 3:** Comparative Analysis of CO_2_ Storage Capacity Per Volume of the Reservoir^a^

			Aperture to size ratio β		
CO_2_ storage capacity per volume [kg/m^3^]			6·10^–2^	3·10^–2^	0.7·10^–2^
Mineralized	Maximum (*h* = *d*_c_/2)	$\frac{M_{CO_{2}}}{V_{c}^{T}}=\frac{1}{{\left(1+\beta \right)}^{2}}\rho_{R}\eta_{\min}\left(\frac{M_{\mathrm{CO}2}}{M_{R}}\right)$	172	182	191
	At shut-off (ε = 0.2)	$\frac{M_{CO_{2}}}{V_{c}^{T}}\approx \frac{2\beta }{\varepsilon }\rho_{R}\eta_{\min}\left(\frac{M_{\mathrm{CO}2}}{M_{R}}\right)$	116	58	14
					
Supercritical	Fracture	$\frac{M_{CO_{2}}}{V_{c}^{T}}\approx 2\beta \rho_{CO_{2}}^{\mathrm{SC}}\eta_{f}$	16	8	2
	Matrix	$\frac{M_{CO_{2}}}{V_{c}^{T}}=\frac{n}{{\left(1+\beta \right)}^{2}}\rho_{CO_{2}}^{\mathrm{SC}}\eta_{m}$	1.2	1.3	1.3
		Total:	17	9	3
					
Dissolved	Fracture	$\frac{M_{CO_{2}}}{V_{c}^{T}}\approx 2\beta \rho_{H_{2}O}{\chi_{CO_{2}}m}_{CO_{2}}\eta_{f}$	5	2	0.5
	Matrix	$\frac{M_{CO_{2}}}{V_{c}^{T}}=\frac{n}{{\left(1+\beta \right)}^{2}}\rho_{H_{2}O}{\chi_{CO_{2}}m}_{CO_{2}}\eta_{m}$	0.3	0.4	0.4
		Total:	5	3	0.9

a Parameters: *P* = 6 MPa and *T* = 50 °C; matrix porosity *n* = 0.1; supercritical CO_2_ density ρ_CO2_^SC^ = 135.2 kg/m^3^; water density ρ_H_2_O_ = 990.6 kg/m^3^; CO_2_ solubility χ_CO_2__ = 0.9 mol of CO_2_/kg water; CO_2_ molar mass m_CO_2__ = 44 g/mol; rock density ρ_R_ = 2900 kg/m^3^. Efficiency coefficients η_min_ = 0.25 for mineralized CO_2_, η*_m_* = 0.1 for supercritical and dissolved CO_2_ in matrix, η*_f_* = 1 for supercritical and dissolved CO_2_ in fractures.

### 3.4 Storage Capacity for Supercritical and Dissolved CO_2_

For comparison, we consider the storage capacity achievable by filling the pore-volume with either supercritical CO_2_^SC^ or CO_2_^aq^ dissolved in water. The fracture volume *V*_c_^f^ [m^3^/m] per unit column height is (Figure 7—Refer to eqs 17 and 24):

$$fracture\,\;volume\,\;\;\;\;\;V_{c}^{f}=V_{c}^{T}-V_{c}^{m}=\frac{\sqrt{3}}{2}\left[{\left(d_{c}+b\right)}^{2}-d_{c}^{2}\right]$$ 26

The CO_2_ mass storage capacity per representative column volume *M*_CO_2__/*V_c_^T^* [kg/m^3^] in supercritical CO_2_ is a function of the CO_2_ density ρ_CO_2__^SC^ at reservoir conditions [kg/m^3^]:

$$in\,\;fractures:\;\;\;\;\frac{M_{CO_{2}}}{V_{c}^{T}}=\frac{{V_{c}^{f}\rho }_{CO_{2}}^{\mathrm{SC}}\eta_{f}}{V_{c}^{T}}=\left(1-\frac{1}{{\left(1+\frac{b}{d_{c}}\right)}^{2}}\right)\rho_{CO_{2}}^{\mathrm{SC}}\eta_{f}\approx 2\beta \rho_{CO_{2}}^{\mathrm{SC}}\eta_{f}$$ 27

$$in\,\;\,the\,\;\,matrix:\;\frac{M_{CO_{2}}}{V_{c}^{T}}=\frac{nV_{c}^{m}\rho_{CO_{2}}^{\mathrm{SC}}\eta_{m}}{V_{c}^{T}}=\frac{n}{{\left(1+\frac{b}{d_{c}}\right)}^{2}}\rho_{CO_{2}}^{\mathrm{SC}}\eta_{m}=\frac{n}{{\left(1+\beta \right)}^{2}}\rho_{CO_{2}}^{\mathrm{SC}}\eta_{m}$$ 28

for a matrix porosity *n* [ ]; the approximation in eq 27 assumes small β. From eqs 24 and 26, the potential storage volume in fractures exceeds the pore volume in the rock matrix *V*_*c*_^f^ >*nV*_*c*_^m^ when the matrix porosity *n* is *n* < ∼2β; however, a proper comparison must consider the efficiency coefficients.

The efficiency coefficients η*_f_* [ ] and η*_m_* [ ] represent the fraction of the fracture and matrix pore space that is effectively occupied by CO_2_. The long-term CO_2_ storage capacity in fractured rock depends on the capillary pressure *P_c_* and the degree of saturation at equilibrium *S_w_*. Fractures, due to their larger apertures, exhibit low capillary entry pressures and allow high CO_2_ saturations even at low *P_c_*, therefore, η*_f_* → 1.0. However, the finer pore structure of the matrix requires higher capillary pressures to be saturated with the nonwetting CO_2_ phase;^91^ hence, the efficiency factor η*_m_* is lower for the matrix.

The same eqs 27 and 28 apply to dissolved CO_2_ by replacing ρ_CO_2__^SC^ with the mass of CO_2_ per unit volume of aqueous solution ρ_CO_2__^aq^ = ρ_H_2_O_χ_CO_2__ *m*_CO_2__, where ρ_H_2_O_ [kg/m^3^] is the density of H_2_O, χ_CO_2__ [mol of CO_2_/kg water] is the solubility of CO_2_ in water at reservoir conditions, and *m*_CO_2__ [kg/mol] is the molar mass of CO_2_ (Note: it assumes that the volume of water does not change with dissolved CO_2_). In this case, the fluid is single phase and both η*_f_* and η*_m_* depend on displacement efficiency.

### 3.5 Comparison

The proportionality between aperture and block size β = *b*/*d_c_* renders the CO_2_ storage capacity independent of the column dimensions in all cases. In fact, these gravimetric-volumetric analyses highlight the central role of mineralogy in defining the CO_2_ storage capacity of a mafic formation: mineralogy determines the extent of thermal contraction during cooling (factor β–Figure 4), the chemical reactions involved in mineralization (eq 19) and the degree of swelling during mineralization (factor ε).

Table 3 compares the anticipated carbon storage capacity through mineralization (full reaction and at shut-off), versus filling the pore space with pure supercritical CO_2_^SC^ or CO_2_^aq^ dissolved in water. Results highlight the important role of volumetric storage in fractures and matrix in short time scales, and the greater role of mineralization in the long term.

Finally, let us compare the predicted storage capacities *M*_CO_2__/*V_c_^T^* summarized in Table 3 with estimates reported in previous studies (or based on reported data): 0.6 kg/m^3^ as supercritical CO_2_ in the Columbia River Basalt Group (computed from ref 56); 27 kg/m^3^ as supercritical CO_2_ and 32 kg/m^3^ as mineralized carbon if all the CO_2_ is fixed as calcite in the Juan de Fuca Plate (computed from ref 92); between 19-and-49 kg/m^3^ storage in calcite form in the Icelandic bedrock;^93,94^ 10-to-1000 kg/m^3^ in mineralized form at the Carbfix site (computed from ref 95) and 212-to-575 kg/m^3^ in carbonate minerals following complete rock reaction in basalt formations in China.^96^ Clearly, volume positive reactions and transmissivity shut-off may significantly reduce some of these high estimates, as discussed above (Table 3).

### 3.6 Discussion — Long-Term Performance

Field data on CO_2_ mineralization within fractured rock masses remain limited due to the scarcity of field-scale studies and the inherent challenges of collecting spatiotemporal data in the field.^97^ As a result, “numerical experiments” that incorporate key geological features (such as mineralogy, fracture statistics and network topology) along with experimentally determined geochemical parameters, offer a critically important framework for investigating the evolution of CO_2_ mineralization in the field.

Multiple lines of evidence support the primary role of fractures in injectivity and storage. Injection wells at the CarbFix site have targeted active faults to exploit fracture-permeability pathways, and there is evidence that vertical columnar jointing results in higher vertical than horizontal permeability.^98–100^ Natural analogues such as mineral-filled vein networks in peridotite formations^101,102^ and laboratory studies confirm preferential mineralization along fracture surfaces.^62,103^ Recent experimental and numerical investigations highlight the importance of fracture characteristics and connectivity in controlling the reaction front and carbonate precipitation.^104^

The precipitation of carbonate minerals during CO_2_-rock interactions can significantly reduce porosity and permeability, and thus contribute to long-term CO_2_ containment.^90,105^ Reactive transport simulations show that mineralization through faults and at fracture intersections can effectively limit leakage and CO_2_ migration.^106,107^

Thick carbonate layers and fully carbonated ancient veins demonstrate the long-term durability of carbonates on geological time scales. However, the thermodynamic stability of precipitated carbonates can be affected by changes in fluid chemistry associated with advective flow regimes or even modified CO_2_ injection protocols. In all cases, assessing “long-term” stability requires a defined frame of reference: for example, gradual leakage occurring several centuries after the transition away from fossil fuels will be less critical than leakage taking place within the coming decades.

## 4 Conclusions

This study explored sequential processes involved in CO_2_ mineralization in mafic basalt formations, from fluid injection to long-term mineralization in the far field. These processes are governed by both mineral composition and fracture network characteristics.

Fractured, fine-grained mafic basalts are excellent candidates for CO_2_ geological storage. Their fracture network is intimately related to mineralogy, formation history and cooling rates. Block size *d_c_* and fracture aperture *b* follow log-normal distributions and both exhibit marked variability, typically σ/μ ≈ 0.4. Aperture is proportional to block size; the ratio β = *b*/*d_c_* varies between β ≈ 0.7·10^–2^ and β ≈ 6·10^–2^.

Dissolution prevails in the nearfield of the injection well. The interplay between Péclet’s and Damkhöler’s numbers anticipates ramified dissolution as the advection velocity diminishes away from the injection well. The initial differences in fracture aperture are amplified by dissolution in the nearfield; however, the hexagonal fracture topology effectively mixes flow at fracture intersections and delays the hydro-chemical positive feedback that causes flow localization.

Mineralization can sequester 0.2-to-0.3 g of CO_2_ per gram of rock. However, because it is a volume-positive reaction, mineralization eventually leads to fracture closure and reduced transmissivity. The volume fraction of reacted rock at shut-off depends on the aperture-to-block size ratio β and the volumetric expansion factor ε, both of which are mineralogy-dependent. The resulting reacted volume fraction at shut-off is given by 2β/ε and may vary between 7%-and-24% of the available matrix volume.

Dissolved CO_2_ in water will occupy the pore space of fractures and the matrix. The mass of aqueous CO_2_^aq^ is a small fraction of the storage capacity achievable through mineralization. Clearly, mineralization offers both high storage capacity and long-term stability.

Mineralogy emerges as the primary governing parameter for CO_2_ storage potential in mafic formations. It determines thermal contraction and fracturing during lava cooling, the chemical reactions involved in mineralization and the degree of swelling that causes transmissivity shut-off during carbon mineralization. Field data together with gravimetric-volumetric analyses prompt the careful reassessment of storage capacity.

## Data Availability

Data compiled in this study are available in the online repository Zenodo.^108^

## List of Notations

*A_f_* [m^2^]: fracture area
*b* [m]: fracture aperture
*C* [mol/m^3^]: concentration (subscripts: *f* = fracture)
*D* [m^2^/s]: diffusion coefficient
*Da* [ ]: damköhler number
*d* [m]: fracture spacing (subscripts: *e* = entablature, *n* = column-normal)
*d_c_* [m]: block size
*H* [m]: column length
*h* [m]: peripheral layer thickness
*i* [m/m]: hydraulic gradient
*K* [m/s]: hydraulic conductivity
*k* [m^2^]: permeability
*L* [m]: fracture length
*M* [kg]: mass (subscripts: CO_2_ = carbon dioxide; R = rock)
*m* [g/mol]: molar mass (subscripts: CO_2_ = carbon dioxide; R = rock)
*n* [ ]: porosity
*P* [Pa]: pressure (subscripts: *c* = capillary; nu = u-node; nu* = u*-node)
*Pe* [ ]: péclet number
*q* [m^3^/s]: flow rate (subscripts: *f* = fractures; *m* = matrix)
*R* [mol/m^2^/s]: reaction rate
*S*_w_ [ ]: saturation of wetting phase
*T* [°C]: temperature (subscripts: *f* = fracturing; amb = ambient)
*t* [s]: time (subscripts: adv = advection; diff = diffusion; react = reaction; *r* = residence)
*v* [m/s]: flow velocity
*V*_c_ [m^3^/m]: volume per unit column height (superscripts: *T* = total; *m* = matrix: *f* = fractures; *r* = reactive)
*V* [m^3^]: volume (subscripts: *f* = fracture; *m* = molar)
*z* [m]: depth
α [°C^–1^]: thermal expansion coefficient
β [ ]: ratio *b*/*d_c_* (=αΔ*T*)
γ [kN/m^3^]: unit weight
δ [ ]: stoichiometric factor relating the amount of rock dissolved per H^+^ consumed
ε [ ]: volumetric expansion
η [ ]: efficiency coefficient (subscripts: *f* = fractures; *m* = matrix; min = mineralized CO_2_)
θ [mol/mol]: CO_2_ trapped in carbonates
κ [m/s]: rate constant
λ [mol/mol]: dissolution factor
μ_d_ [Pa·s]: dynamic viscosity
ρ [kg/m^3^]: density (subscripts: SC = supercritical CO_2_; aq = aqueous CO_2_; R = rock)
τ [m^2^/s]: fracture transmissivity
χ_CO_2__ [mol CO_2_/kg water]: CO_2_ solubility
φ [mol/mol]: carbonate formed with each cation
ψ [mol/mol]: cation fraction in rock
